# Public participation: healthcare rationing in the newspaper media

**DOI:** 10.1186/s12913-022-07786-w

**Published:** 2022-03-28

**Authors:** Audun Brendbekken, Bjarne Robberstad, Ole F. Norheim

**Affiliations:** 1 Department of Global Public Health and Primary Care, Bergen Centre for Ethics and Priority Setting (BCEPS), University of Bergen, Årstadveien 21, 5020 Bergen, Norway; 2 Department of Global Public Health and Primary Care, Health Economics, Leadership and Translational Ethics Research (HELTER), University of Bergen, Årstadveien 21, 5020 Bergen, Norway

**Keywords:** Participation, Deliberation, Priority setting, High-cost, Norway, Media, Rationing

## Abstract

**Background:**

It is impossible to meet all healthcare demands, but an open and fair rationing process may improve the public acceptability of priority setting in healthcare. Decision-making is subject to scrutiny by newspaper media, an important public institution and information source for discussions about rationing. In Norway, healthcare rationing has been subject to public debate both before and after the establishment of “The National System for Managed Introduction of New Health Technologies within the Specialist Health Service” (New Methods) in 2013.

**Aim:**

To describe and assess the development of the public debate on Norwegian healthcare rationing through three cases in print media.

**Methods:**

We purposively sampled Norwegian newspaper articles between 2012 and 2018 concerning three reimbursement decisions in the Norwegian system. The reimbursement decisions were ipilimumab (Yervoy, *n* = 45) against metastatic melanoma, nivolumab (Opdivo, *n* = 23) against non-small cell lung cancer, and nusinersen (Spinraza, *n* = 68) against spinal muscular atrophy. Cases were analysed separately using the qualitative method of systematic text condensation.

**Results:**

Our analysis highlighted four common themes—money, rationales, patient stories, and process—and a unique theme for each case. Ipilimumab was uniquely themed by rationing rejection, nivolumab by healthcare two-tiering, and Spinraza by patients’ rights. We found wide media deliberation among a multitude of stakeholders in all cases. Perceptions of rationing were found to be chiefly aligned with previous empirical research. We found that the media reported more frequently on opposition to rationing compared to findings from previous studies on Norwegian healthcare decision-making attitudes. We think this was influenced by our selection of cases receiving extraordinary media attention, and from media sources being subject to political communication from special interest groups.

**Conclusion:**

We observed that the introduction of New Methods institutionalised Norwegian healthcare rationing and isolated the public debate into conversations between stakeholders and decision makers outside the political sphere. The findings from these three extraordinary debates are not generalisable and should be seen as a stakeholder learning opportunity regarding media coverage and engagement with expensive specialist healthcare decision-making in Norway.

**Supplementary Information:**

The online version contains supplementary material available at 10.1186/s12913-022-07786-w.

## Introduction

Finite governmental healthcare resources are under increased pressure due to rising healthcare demands and medical technology innovations [1, 2]. Against the insatiability of healthcare demands, public deliberation and fair processes may help legitimise priority-setting institutions [3]. However, when specific interests conflict with allocation decisions, processes, and outcomes may be subject to scrutiny by the public media [4, 5].

### Norwegian context and rationing institutions

Fair healthcare rationing is envisioned in public policy as paramount for Norway to retain its egalitarian welfare state [6, 7], see Table 1. Norwegian healthcare rationing was revised through three national committees on priority setting in 1987, 1997, and 2014 [7, 10, 11]. In 2016, a governmental white paper formulated the specialist healthcare priority-setting criteria as (i) health benefit, (ii) resource use, and (iii) severity of disease [7, 9, 12], see Table 1.

**Table 1:** Norway and Norwegian healthcare distribution

• Norway had 5.3 million inhabitants and its GDP/capita was US$63,293 in 2019 [8] • Norwegian healthcare is tax-funded with yearly co-payment up to a ceiling of US$280 • Three priority-setting criteria regulating access to specialist healthcare: [9] o Health benefit: Priority of intervention increases with its expected benefit. o Resource use: Interventions requiring fewer resources are prioritized o Severity of disease: Priority of intervention increases according to the severity of the condition.	

In 2013, “The National System for Managed Introduction of New Health Technologies within the Specialist Health Service” (New Methods) was established. New Methods is a system which regulates public access to new medical technologies within the specialist health service [13]. The regional health authorities (RHAs) hold a dual responsibility for specialist healthcare services and financing through their responsibility for New Methods [14]. All stakeholders are free to suggest technologies, but proposals are commonly made by Norwegian hospitals, pharmaceutical manufacturers, and the Norwegian Medicines Agency. Proposals are administered by the Ordering Forum, which comprises the technical directors of the four RHAs and one observer [13]. The Norwegian Medicines Agency conducts single technology assessments (STAs) for proposed medicines and the Norwegian Institute of Public Health conducts STAs or full health technology assessments (HTAs) of proposed technologies on behalf of the Ordering Forum [15]. At a local level, hospital clinicians trained by their RHAs may perform mini-HTAs to evaluate and introduce new, limited technologies but not medicines into clinical practice [15]. Approved technologies are considered for implementation on the basis of HTAs by the Decision Forum, which comprises the Chief Executive Officers (CEOs) of the four RHAs with one representative from user organisations and one from the Directorate of Health [15]. Their decision-making is based on HTAs and according to national priority-setting criteria (see Table 1). The Decision Forum is mandated to remain neutral with regards to patient group size, age and diagnosis [9]*.* The Decision Forum often negotiates undisclosed prices with manufacturers, and their meetings are always closed to the public [16]. Meeting notices, case documents and protocols are publicly available with commercial details redacted. Their decisions are regularly subject to scrutiny by the public media.

### Priority setting and the public

Against the insatiability of individual demands, public deliberation to resolve moral disagreement is widely acknowledged as a source of political legitimacy [5, 17–20]. In healthcare, the often-cited normative framework accountability for reasonableness (A4R) requires legitimate rationing processes to meet the four criteria of (i) publicity, (ii) relevance, (iii) appeals and revision, and (iv) regulation [3]. A4R leaves disagreements about substantive justice to be resolved through deliberation, which does not ensure moral legitimacy in an imperfect society.

Fair, open deliberation is not without its challenges in a pluralistic society with power asymmetry and institutional vulnerability [3, 19–21]. Public discourse and media debate may be driven by political communication, special interests, or editorial misinterpretations [21]. Fear of media scrutiny may be an obstacle to unpopular decision-making [7, 22]. Patients rely on news media for healthcare rationing information [23], which oftentimes is unnuanced and unconstructive regarding priority setting [24]. Media coverage influences public trust and understanding of priority setting [25] and engages stakeholder action [26]. Without an explicit process, media-driven or other forms of implicit rationing may lead to unjust allocation between loud winners and silent losers [7, 12, 27].

Healthcare rationing has traditionally been subject to wide deliberation in Norwegian newspapers [28], but debate also occurs outside print media. Norwegian newspaper media are seen as an important institution in modern society whereby the communication of resource allocation bears normative importance to the legitimacy of the welfare state [4, 29]. Central health institutions, such as New Methods, the Norwegian Institute of Public Health, and the Health Directorate, are themselves active communicators of healthcare decisions through television debates, regular press conferences, and interviews.

In Norway, empirical studies on rationing have analysed procedural and substantive justice. A study of two Norwegian treatment allocation decision cases from 2005 concluded that Norwegian drug reviews lacked sufficient regulation to ensure procedural transparency [27]. A web-based survey from the Norwegian Citizen Panel revealed that 47% of 1620 respondents believed current healthcare resource usage was insufficient to secure full preparedness and supply of useful interventions, and 50% believed that Norway could afford to offer all useful health measures and interventions without neglecting other societal tasks [30]. After the introduction of New Methods, oncologists experienced distrust with the centralised drug review process and reported a lack of engagement with the process, disagreement with rationing criteria, and a lack of process transparency and appeals [16]. Public participation is viewed, especially by patient organisations, as essential to procedural legitimacy, yet entangled by vocal patient interests, political stakeholders and media demands [31]. Stakeholders and decision makers largely agree with national guidelines for priority-setting criteria [9], although stakeholders’ prioritisation of equity may be stronger than in recent policy proposals [31, 32].

It is important to study rationing in newspaper media because print media offer an edited platform for public debate on healthcare rationing. Such an analysis may provide a learning opportunity about which stakeholders participate in the public debate and the extent of their agreement with the rationing process and decision outcomes.

To our knowledge, no qualitative studies have described the public debate on Norwegian healthcare rationing utilising newspaper coverage.

This article describes and assesses the development of the public debate on healthcare rationing through three cases in Norwegian print media.

## Methods

### Study design

In this qualitative study, we used systematic text condensation (STC) [33] to describe three Norwegian healthcare rationing debates. The data material consisted of selected Norwegian newspaper articles presenting statements and reactions concerning three healthcare rationing debates between 2012 and 2018. STC is a pragmatic analytical approach [33] also suitable for cross-sectional thematic analysis of newspaper articles [34]. Although A4R is a normative framework for legitimacy, stating a benchmark of acceptability for priority setting and procedural justice [3], we used it as a substantive theory to focus our inductive data analysis [33–36] because of its wide endorsement in the literature on priority setting and in Norwegian healthcare rationing practice and empirical studies [7, 16, 27, 37, 38].

### Data material

We selected data from newspaper coverage concerning three reimbursement decisions between 2012 and 2018 in the Norwegian system. The chosen reimbursement decisions were ipilimumab (Yervoy, *n* = 45) against metastatic malignant melanoma, nivolumab (Opdivo, *n* = 23) against non-small cell lung cancer, and nusinersen (Spinraza, *n* = 68) against spinal muscular atrophy, as described in Tables 2, 3 and 4. Reimbursement decisions are hereafter designated by their most frequently cited names in the media: ipilimumab, nivolumab, and Spinraza.

**Table 2:** Context to the ipilimumab debate

Ipilimumab as second-line treatment against inoperable metastatic malignant melanoma was first evaluated in 2012, prior to the implementation of New Methods. The Norwegian Medicines Agency initially estimated the cost-effectiveness to be approximately US$145 K/QALY, substantially higher than the perceived existing willingness to pay for health [39]. Before New Methods, the National Council for Quality Improvement and Priority Setting in Health Care made transparent and evidence-informed recommendations on the implementation of specialist healthcare treatments of principal interest. Recommendations were given to the Directorate of Health, which then decided on implementation together with the bodies responsible for budgeting, that is, the Regional Health Authorities [40]. For ipilimumab, the Norwegian Medicines Agency produced a single technology assessment [39], while the Directorate appointed an advisory expert panel to evaluate new cancer drugs. The panel advised against implementation in April 2012 [41], before medically recommending implementation in January 2013. In March 2013, the Directorate decided against implementation due to cost-effectiveness considerations [42]. Three days later, the Minister of Health intervened on the decision and allocated the equivalent of about US$18 M in research funds for a nationwide phase IV-study, which, in practice, implemented the drug for the entire patient group [43]. Simultaneously, the minister announced the creation of the third committee on priority setting in healthcare. New Methods was introduced later in 2013, and ipilimumab was implemented as standard therapy for the metastatic malignant melanoma patient group in October 2014 [44, 45].

**Table 3:** Context to the nivolumab debate

Nivolumab as second-line treatment against non-small-cell lung cancer (NSCLC) of both squamous and non-squamous cell carcinoma was evaluated by New Methods in 2015. The initial incremental cost-effectiveness estimate of nivolumab compared to the standard of care for NSCLC patients was approximately US$175 K /QALY according to the Norwegian Medicines Agency, and 1150 patients were potentially eligible [46, 47]. The Decision Forum decided against implementing nivolumab twice in 2016 [48]. After tendering, the Decision Forum implemented a cheaper option, pembrolizumab (Keytruda), for the same indication in September 2016 [49].

**Table 4:** Context to the Spinraza debate

Spinraza as treatment for spinal muscular atrophy (SMA) was first evaluated by New Methods in 2017. The Norwegian Medicines Agency estimated the cost-effectiveness to be between US$2 M and 5 M/QALY depending on SMA subtype severity [50]. Spinraza was the first drug with any effect against the severe and rare degenerative neuromuscular disease SMA, and no patients could afford it privately. After the European Medicines Agency approved Spinraza in spring 2017 [51], Oslo University Hospital and the manufacturer, Biogen, provided free treatment to 10 Norwegian children with the severest subtype, SMA-1. In October 2017, the Decision Forum granted continued treatment for these patients, but denied treatment for other patients, thrice between October 2017 and February 2018, whilst the Decision Forum haggled for a better price. In February 2018, Spinraza was implemented for nonadult patients dependent on eligibility criteria [52], as clinical evidence only existed for minors [53]. Spinraza is currently being evaluated for adult patients [54].	

We chose these three cases because of their suitability for describing and assessing Norwegian healthcare rationing debates in newspaper media before and after the introduction of New Methods. The cases shared a similar pattern of initial refusal to implement expensive treatment, followed by a public discussion that culminated in some compromise to meet the afflicted patient group’s needs. The material spans the operationalisation of New Methods in 2013 and the white paper implementation of the new priority-setting criteria in 2016. It should be noted that ipilimumab and Spinraza represented extraordinary public discussions in Norway.

We purposively sampled relevant and varied data to give sufficient informational power to our broad study aim [55]. We collected data from Atekst [56], the online search engine for Norwegian newspapers, by creating a search algorithm incorporating the disease and the generic and commercial drug names for each case, see Additional file 1. We selected nine newspapers available online for data sampling based on article frequency and outreach variance, meaning that our data material was widely accessible to large parts of the Norwegian population. Amongst the selected national newspapers Dagbladet and Verdens Gang are Norway’s most read papers with a somewhat tabloid profile, whereas Aftenposten is considered more broadsheet and holds Norway’s largest newspaper subscriber base. The selected regional newspapers Fædrelandsvennen and Bergens Tidende cover southern and western Norway and are both accessible by subscription. We selected one healthcare periodical, Dagens Medisin based on its central position in the Norwegian specialist healthcare debate. Three local subscription-based newspapers were selected after a pilot search revealed that Hallingdølen, Saltenposten and Romerrikes Blad respectively covered the ipilimumab, nivolumab and Spinraza cases more frequently than any other local newspapers. This produced an overall hit of 107 articles for ipilimumab, 64 articles for nivolumab, and 120 articles for Spinraza. We read through all and selected newspaper articles for analysis based on the texts’ relevance to the study question. Relevant newspaper articles contained some stakeholders’ statements or reactions to the chosen allocation decision, including statements made by the deciding health authorities. Purely descriptive notices were excluded from the material. We sampled our data material in October 2019, and its source distribution is shown in Table 5.

**Table 5:** Sampled articles and newspaper distribution across cases

Newspaper	Ipilimumab	Nivolumab	Spinraza
National: Verdens Gang, Dagbladet, Aftenposten	19	2	27
Health technical: Dagens Medisin	10	10	10
Regional: Bergens Tidende, Fædrelandsvennen	5	8	12
Local: Romerrikes Blad, Saltenposten, Hallingdølen	11	3	19
Total articles	**45**	**23**	**68**
Timespan articles	2012–2017	2015–2017	2017–2019

### Data analysis

**Fig. 1:**
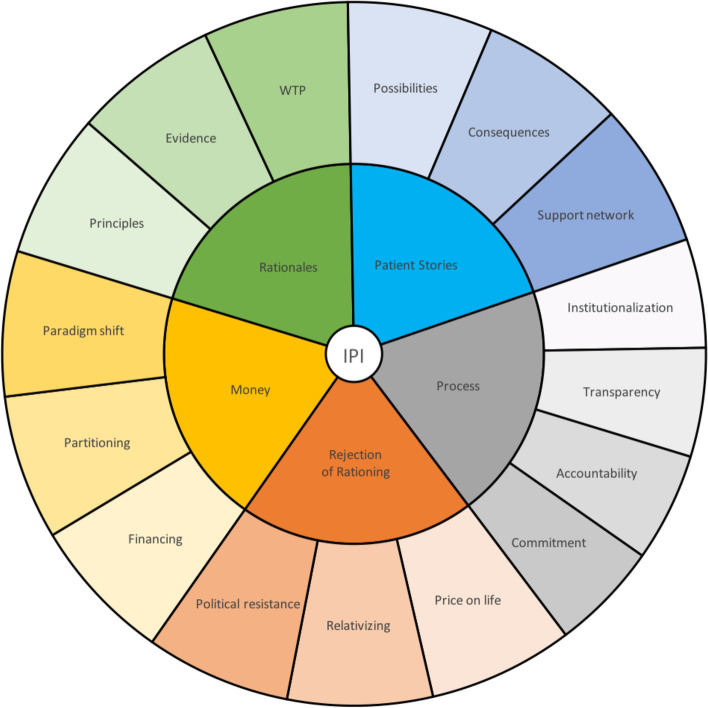
Ipilimumab (=IPI) codes (inner) and subcategories (outer) from analysis

**Fig. 2:**
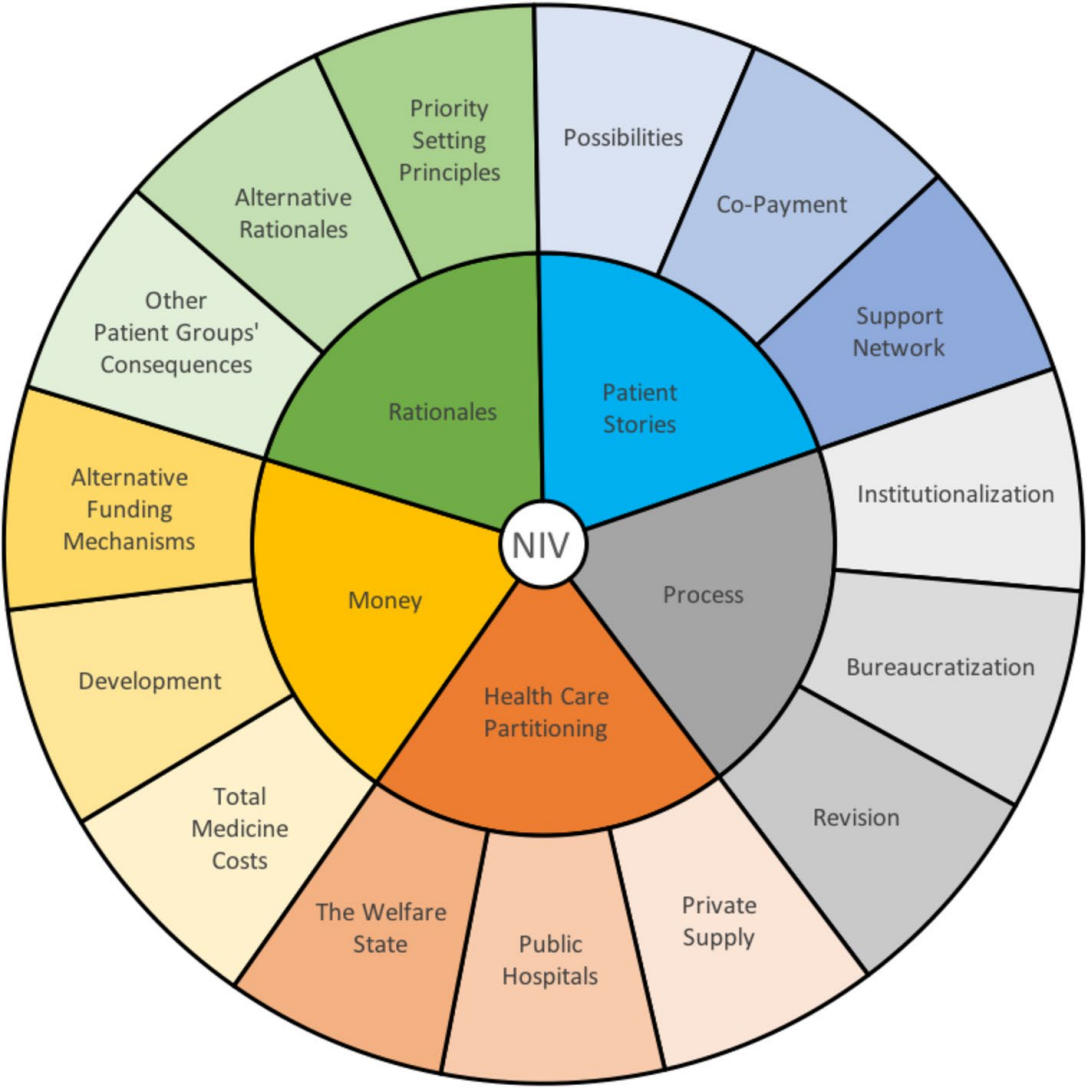
Nivolumab (=NIV) codes (inner) and subcategories (outer) from analysis

**Fig. 3:**
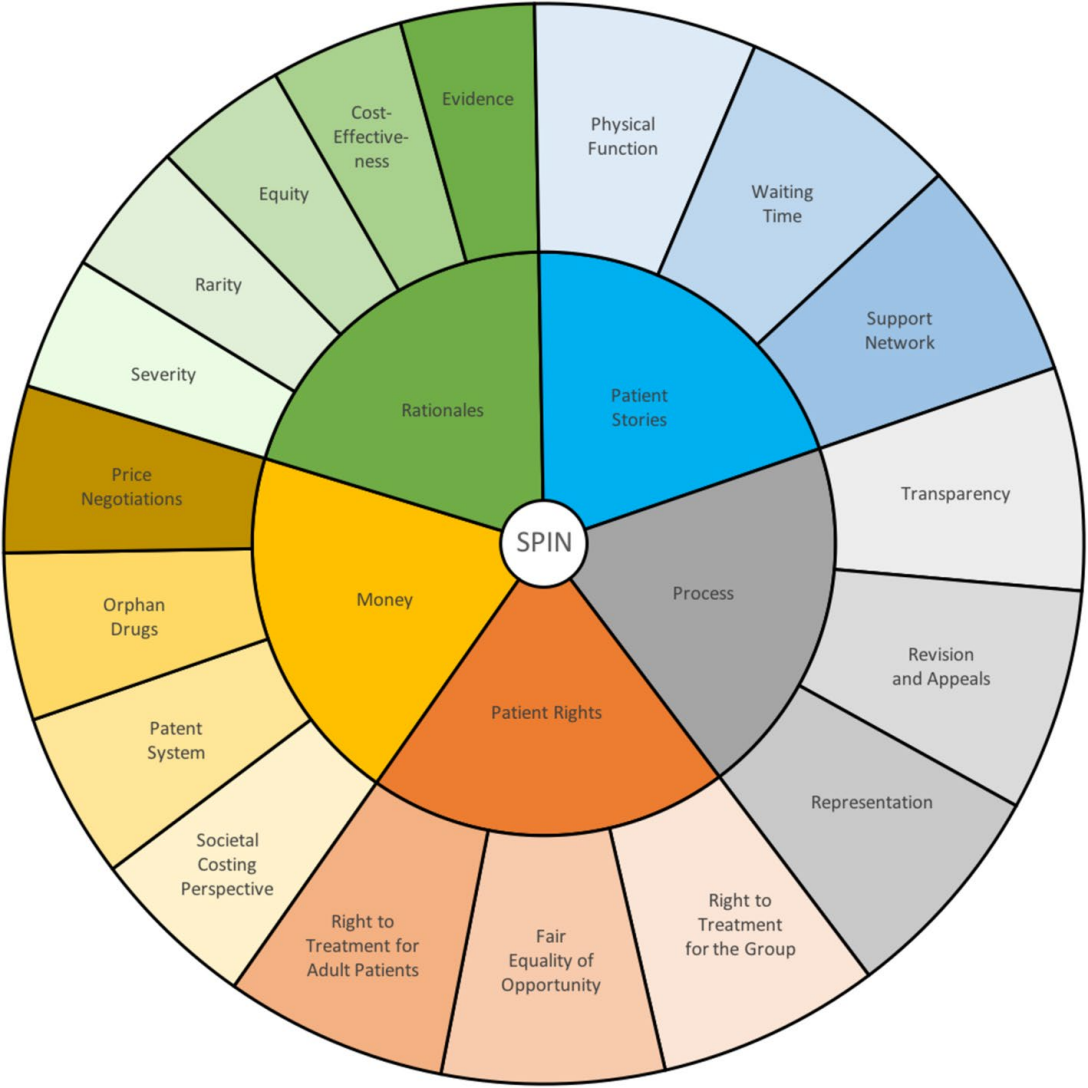
Spinraza (=SPIN) codes (inner) and subcategories (outer) from analysis

For data analysis, we applied STC, which is suitable for developing new descriptions about a studied concept, such as healthcare rationing, in the newspaper media [33]. We followed the four sequential but iterative analytical steps of STC by (i) thematic ordering of total impressions; (ii) identification and sorting of meaning units; (iii) condensation of meaning; and (iv) synthesising descriptions [33]. We conducted the STC on each case separately but in parallel. Throughout, we made analytical comparisons between the cases and iteratively revised some analytical decisions, as informed by a deeper understanding of the material. The analytical steps (ii) and (iii) for each case are shown in Figs. 1, 2 and 3. The reported analytical themes in Figs. 1, 2 and 3 emerged from the data due to their frequency, similarity between cases and relevance to the study question.

Our use of A4R to narrow analysis and its normative standards for legitimate priority setting influenced our preconceptions by enhancing our attention to its criteria of publicity, relevance, revision, and regulation for procedural justice in analysis [3, 57]. We used A4R as a substantive theory to sharpen the focus of our inductive analysis and provide a subject-specific perspective on our data material [33–36]. We remained aware of A4R’s relevance to the study question and data selection but allowed the data analysis to develop on its own terms. We endeavoured to maintain an awareness of the topics in the data not explicitly related to A4R but central to rationing. By carefully reading the newspaper articles and their distinct parts, we carved out various nuances of the rationing debate, which were themed in a larger context.

We applied a data editing style as formulated by Crabtree and Miller [58], allowing themes and categories in the data to develop on their own terms rather than being predetermined by our background understanding of A4R as a substantive theory [3, 35].

We performed a cross-cutting results comparison between the three cases. Finally, to assess the development of the Norwegian healthcare rationing debate, empirical findings informed theoretical reflection by A4R and theories of deliberative democracy [3, 5, 17–20].

## Results and findings

We selected newspaper articles on reimbursement decisions regarding ipilimumab (*n* = 45), nivolumab (*n* = 23) and Spinraza (*n* = 68) for analysis. The three cases, as explained in Tables 2, 3 and 4, portray healthcare rationing at different periods during the development of New Methods. Analytical steps, as shown in Figs. 1, 2 and 3, revealed four central, common themes in all cases—money, rationales, patients’ stories, and procedural concerns. Additionally, we found distinct discussions of rationing aversion during ipilimumab, two-tiering during nivolumab, and patients’ rights during Spinraza. In what follows, we report the main findings of our analysis.

### Ipilimumab (Yervoy) against metastatic malignant melanoma

Ipilimumab was characterised by decision-making fragmentation, as New Methods was not yet in place and rationing responsibility was shared between several institutions (see Table 2). The results concern new challenges to the funding scheme, disputed willingness to pay, patients fighting for treatment, New Methods taking over a fragmented process, and rationing aversion.

#### Challenges to the funding scheme

While several editorials and statements by oncologists praised ipilimumab as a paradigm shift in cancer care, some also expressed concerns that the expensive and sought-after drug posed a challenge to the health services’ funding structure. Industrial leaders, editorial writers, and oncologists also warned that poor public willingness to pay could invoke a two-tiering of cancer care. Concerning treatment funding, some editorials suggested introducing a cancer fund or user fees, while some oncologists worried about funding after completion of a national phase IV-study introduced by the minister of health that temporarily implemented ipilimumab for the patient group.

#### Disputed willingness to pay

Rationales for implementation changed with new evidence and political will. Initially, the expert panel advised that, due to limited evidence and the intervention not being cost-effective, ipilimumab should not be implemented. Leading oncologists, being positively inclined to the introduction of ipilimumab, criticised the advice, saying that “patients should of course receive both palliation and life-prolonging treatment”. This was backed by a citizen who criticised the quality-adjusted life-year (QALY) logic for discriminating against terminally ill patients:

“When the Directorate anchors its decision to refuse vital treatment in this logic, it assumes an unnecessarily cruel recipe for health discrimination” [59]. (Verdens Gang, 28 March 2013)

The expert panel revised their medical advice to a recommendation for implementation after considering new and improved evidence. However, the cost-effectiveness ratio was still higher than the Directorate of Health’s willingness to pay; the Directorate stated its commitment to national priority-setting criteria and declined ipilimumab. Later, after the ipilimumab study was introduced, one technical director of a regional health authority said that the willingness to pay was effectively doubled.

#### Patients fight for treatment

Patients who were hoping for life-prolonging treatment experienced a life-and-death struggle with the health authorities for access to medicine but celebrated the news of the national study as a victory. During the fight, one patient said declining ipilimumab was equal to “euthanasia”, and that they were forced to become “health refugees” after a long life of paying taxes. In their battle for treatment, patients received public support from their oncologists, several editorials, members of the political opposition in Parliament, and online petitions demanding access to treatment. One patient who had been particularly vocal about the need for treatment was proud to have been instrumental in the expert panel’s eventual decision to recommend treatment:

“Had I not brought up the issue, perhaps this would have never happened. So, I am proud for giving others the opportunity to also try” [60]. (Romerrikes blad, 29 February 2013).

#### New methods take over fragmented process

Coverage of the ipilimumab decision-making process portrayed a weak and fragmented system. The Directorate of Health participated in secret price negotiations but declined an undisclosed offer for ipilimumab so as not to harm public trust in the system. Subsequently, the minister of health used his “unique appeals right” and intervened to fund a nation-wide ipilimumab study. This was called a necessary retreat by several editorials, who also criticised the director general for neglecting the expert panel’s advice. However, one editorial said this critique was a misunderstanding, as the director, unlike the expert panel, could not ignore costs. In the aftermath of the ipilimumab spectacle, one commentator noted that the rationing process had been fragmented and lacked political legitimacy:

“Technical experts guarding the gates for treatment today, are disappointed with politicians after this spring’s controversy. They demand – and likely deserve – stronger support for their difficult decision-making” [61]. (Aftenposten, 7 November 2013)

#### Rationing aversion

The public debate in “the world’s richest country” bristled with an aversion to rationing. As patients and professionals argued that comparable countries already used ipilimumab, one physician concluded that lack of access was simply a result of “the economic politics”. Furthermore, there was wide opposition to “putting a price on life” and a pharmaceutical leader called rejecting ipilimumab an “admission of failure for Norwegian healthcare egalitarianism”.

### Nivolumab (Opdivo) against non-small cell lung cancer

As some patients privately purchased nivolumab while New Methods tendered for lower procurement costs, discussion circled on privatisation of cancer care (see Table 3).

The nivolumab results relate to fiscal concerns, displacement of other treatments, out-of-pocket payment, unpreparedness of New Methods, and two-tiering of healthcare.

#### Fiscal concerns

The implementation of nivolumab posed a threat to hospitals’ budgets. Members of the Ordering Forum warned that the high cost for a large patient population could break hospital budgets, saying that immunotherapies pose tough future rationing dilemmas. Oncologists recognised the financing problem in the immunotherapeutic paradigm shift but thought the price for nivolumab was “absurd” because its wide indication for a large patient population should indicate a lower price per patient. The financing problem barred patients from treatment during tendering. To reduce waiting time for patients, oncologists and members of industry proposed implementing a cancer fund to provide temporary financing. Some physicians warned against creating a fund only for cancer patients or allowing industry to dictate financing, saying there was a need instead for objective price-setting mechanisms based on disease severity and frequency, consequences of not using medication, R&D costs, and private and public willingness to pay.

#### Displacement of other treatments

Despite its efficacy, the expenditure of nivolumab was considered to be worrisome. New Methods stated a commitment to a diagnosis-neutral, effective, and fair allocation of healthcare resources. The Decision Forum decided to reject nivolumab, as it was not considered cost-effective and therefore would displace other essential treatments. The decision-making rationales were publicly disputed, and some oncologists argued that lung cancer patients should receive nivolumab because it had been provided to other cancer patient groups and was documented as highly effective for 20% of the lung cancer patient group. After some patients purchased nivolumab privately, one clinical ethical committee ruled that oncologists were not allowed to administer privately purchased treatment within public hospitals, as this would violate the principle of equal treatment. Reflecting on the debate, one Decision Forum member highlighted the problem of opportunity costs:

“The challenge is to finance new medication without affecting other patient groups. (…) but it is inescapable that implementation will affect others” [62]. (Dagens Medisin, 12 January 2017)

#### Out-of-pocket payment

Patients who personally paid for nivolumab expressed that the wait for public hospital drug approval exacerbated societal class divisions. To afford medication, one patient sold his boat, and the relatives of another raised money through Facebook. A public hospital oncologist who despaired that he was unable to help his patients said that nivolumab brought them hope for a longer and better life. This hope was reinforced by stories from some patients who had experienced the wonders of shrinking lung tumours after using nivolumab. One patient was admittedly bitter about not receiving medication:

“The drug could have given me more time. I have paid taxes for 43 years and never demanded anything. (…) Why must we scrape and beg, when this is something we are entitled to? The politicians are nothing but assassins” [63]. (Bergens Tidende 28 October 2015)

#### Unpreparedness of new methods

During the nivolumab debate, New Methods faced complaints about treatment delay, judicial incapacitation, and inefficiency. Some physicians warned that the Decision Forum’s evaluation time and tendering obstructed treatment opportunities for terminally ill patients and led to a two-tiering of cancer care. Furthermore, one oncologist said that the Decision Forum members were judicially incapacitated, as they lacked medical competence and focused only on cost containment when rationing. Other physicians said there was an urgent need for objective price-setting mechanisms and better predictive biomarkers. Critiquing the slow process, an industry director said that New Methods was unprepared for the cancer revolution and should streamline their drug procurement negotiation process. Still, the leader of the Decision Forum said he experienced greater public acceptance for priority setting in healthcare in the wake of the nivolumab debate.

#### Two-tiering of healthcare

The lack of public access to nivolumab instigated a two-tiering of lung cancer care that some oncologists deemed a breach of the promises of the welfare state. Oncologists said Norway, compared with other nations, was late in providing immunotherapy and voiced a concern that it would require political courage to both fight supranational monopolies and avoid a pariah state and private elite within healthcare, as equitable treatment is a moral imperative. An editorial, however, claimed that it was a political responsibility to achieve public acceptance for priority setting grounded on technical advice. Meanwhile, private hospitals and insurers experienced a huge demand from rich Norwegians. Both public and private hospital oncologists said that they experienced the situation as medically and ethically uncomfortable. One public hospital oncologist said the low willingness to pay for lung cancer patients was unethical and incompatible with solidarity:

“The Decision Forum and [minister of health] can defend the “need for priority-setting”, but this is a choice of values, an ethical choice. Do taxes no longer qualify for a valid social contract where each pays according to their ability and receives according to their needs?” [64]. (Aftenposten 17 August 2016)

### Nusinersen (Spinraza) against spinal muscular atrophy (SMA)

The Spinraza discussion crystallised the trade-off between the patient’s right to essential treatment and the priority-setting problem (see Table 4). Results revolve around price negotiations, balancing weights for a rare disease, waiting and hoping for treatment, secretive and unapproachable decision makers, and patient rights violations.

#### Price negotiations

Accounts of procurement portrayed unwavering bargaining. The Norwegian Medicines Agency named Spinraza as “the world’s most expensive drug”, and the Decision Forum said “yes to Spinraza” but “no to an unethical price” during three rounds of price negotiations. One health economist saw the reference to unethical pricing as “a strategy by pressured [decision-makers]”. Throughout, the minister of health attempted to co-negotiate with Nordic countries for a lower price, but without success. Decision-makers’ stance against “soaring costs” was initially applauded by most editorials, while some physicians and activists called the patent system dishonest and unfair. However, as patients’ parents and the advocacy organisation SMA Norway criticised the “greedy” health authorities for “playing to the gallery”, the editorial tide turned against the Decision Forum during price negotiations. Arguments from commentators and SMA Norway addressed societal and economic gains of patients improving from treatment. Meanwhile, the Norwegian Parliament narrowed the legal definition of orphan disease, leading paediatricians and SMA Norway to fear that drug reform could challenge the financing of orphan drugs. Paediatricians, members of New Methods, and some editorials all agreed that Spinraza represented a treatment revolution and the first of many priority-setting dilemmas for orphan drugs, and the Decision Forum called it “the hardest case [they had] ever evaluated”. One editorial worried about future debates:

“Let's hope the next Spinraza-case won’t come in a long time. Because these cases only have losers: they are a mockery to patients and their parents, and function like pure toxins in the relationship between the pharmaceutical industry and the health authorities” [65]. (Dagens Medisin, 9 November 2017)

#### Balancing weights for a rare disease

Substantive disputes concerned the relative weighting of equitable treatment for a severe and rare disease against an expensive treatment, with evidence pertaining only to minors. Initially, some editorials and physicians accepted the Decision Forum’s refusal, saying that “utility must be the guiding star”. One Ordering Forum member formulated the dilemma between the severity and resource use criteria as follows:

“[T]his concerns children and a disease without alternative treatments. Parliament (…) said that consideration for children weigh heavy, and that [New Methods] may consider patient group size. However, equivalent treatments for other small patient groups will arrive. If we accept Spinraza, we must also accept these (…), then there will be fewer resources for [health priorities] like drug addiction and psychiatry” [66]. (Bergens Tidende, 23 October 2017)

Reflections on this issue were found in the statements of several paediatricians, editorials, and the minister of health, who said there could not be an infinite willingness to pay for rarity. Arguing for implementation, patients and their parents challenged the legality of only treating a subgroup of patients. Supported by UNICEF, SMA Norway called it “boundlessly unethical” to categorically refuse orphan drugs for a few vulnerable children who would have a minor impact on the health budget.

After minors were granted access, one ethicist concluded that “implementation had been hard due to the low level of cost-effectiveness”. The age limit was supported by paediatricians and members of Parliament by reference to evidence for minors but condemned by patients and their representatives who disputed that evidence was lacking and claimed the Decision Forum violated the principles of equitable treatment, individual consideration, and age neutrality in cases involving adult SMA patients.

#### Waiting and hoping for treatment

Patients waited and hoped for access to a drug that could slow disease development and yield improvements in motor skills, ability to speak and strength. Waiting anxiously, parents feared that their children with SMA would live in suffering and face an early death without medication. During extensive negotiations, disillusioned patients became “sick of waiting” and felt as if they were being held hostage in a battle between the health authorities and the industry. Throughout, patients supported each other in a Facebook group #JAtilspinraza, and some attempted to raise funds for medication for individual children. The news of implementation was bittersweet, with the relief for young patients and their parents being overshadowed by the despair of adult patients still lacking access.

#### Secretive and unapproachable decision makers

As negotiations took time, decision makers faced criticisms of secrecy with respect to confidential drug prices, representation within the decision-making process and unapproachability. When the Decision Forum signed an undisclosed price deal, physicians said that cost effectiveness remained unknown but that strict eligibility criteria indicated an unusually high price. Criticising price secrecy, one lawyer said that patients may demand transparency if denied access to medicine.

Concerning representation, paediatricians who had contributed to the Spinraza negotiations expressed a desire to participate systematically in future orphan drug method evaluations. In addition, young patients were happy to meet the Decision Forum and express their need for medication. However, one geneticist claimed that the media had become the natural appeals institution. Another story covered a governmental proposal to exempt the Decision Forum from a regulation requiring open pricing and a mechanism of complaint. Several lawyers and health professional unions were sceptical of this proposal and called the Decision Forum an already opaque concentration of power violating the rights to individual consideration and appeals. One lawyer and patient parent criticised the Decision Forum as being judicially incapacitated:

“They make decisions that in the worst case become a death sentence for patients, without justification or possibility of appeal, loosely grounded in an inaccessible body of regulations” [67]. (Verdens Gang, 13 February 2018)

#### Patient rights violations

For a vulnerable patient group lacking options, the paramount argument was their right to essential healthcare. One parent of a patient called Spinraza a struggle to “create an alternative future” for Norwegian patients who saw comparable countries using treatment they would not get access to, which in turn made them more ill. One patient expressed that they experienced the lack of access to this treatment as equal to “a wish for their death” [68]. As negotiations persisted, the patient organisation SMA Norway threatened to sue and demanded immediate access to the only proper treatment for a severe condition, parallel to the ongoing price negotiations. Their demand was anchored in the constitution, patient law, and the UN Children’s Convention with support from a UNICEF Norway lawyer and spokesperson [69].

After implementation, some adult patients and citizens called the age limit an inhumane and judicially unacceptable breach of the Patient’s Rights Act [70] and human rights. Following that, SMA Norway filed a lawsuit against the Norwegian government on the grounds of age discrimination, but this was later retracted.

## Discussion

To our knowledge, this is the first study to use newspaper coverage to assess public participation in the Norwegian healthcare rationing debate. We found wide media deliberation in all three allocation cases and more opposition to healthcare rationing in print media than in previous empirical survey results. We observe that following the establishment of New Methods in 2013 the public debate was less politicised. The ipilimumab case, considered before New Methods, was highly politicised in the media as the minister of health intervened against the decision by the Directorate of Health and offered treatment through a nationwide study. The ipilimumab process was also criticised for lacking political legitimacy [61]. The public debates regarding the more recent cases of nivolumab and Spinraza, considered by New Methods, were characterized by far less political involvement. Below, we elaborate on our findings and the strengths and limitations of our study design.

### What is previously known and what does this study add?

Our findings are largely consistent with empirical studies of Norwegian priority-setting perceptions, as both physicians and decision makers publicly accept explicit priority setting, although clinicians criticise New Methods for inequitable decision-making and for lacking transparency and clinical representation [16, 30–32].

We found that there was more opposition to healthcare rationing among stakeholders in the studied cases than previously reported in empirical studies [30–32]. Budget restriction critiques were frequently raised by patients, their representatives and physicians, editorials and other stakeholders and interpreted as opposition to rationing healthcare. Newspaper articles are, unlike interviews or surveys, publicly available and edited information sources subject to political communication and interest group pressure [4, 21, 32]. The power of newspapers to engage stakeholders and influence public trust in healthcare rationing [22–26] may partially explain how all three debates culminated in some treatment provision for affected patients.

In all cases, we found wide media deliberation. Patient representatives who advocated implementation were supported by clinicians and pharmaceutical industry leaders, and sometimes by opposition political leaders. Newspaper editorials initially supported cost-effectiveness considerations in all cases, and in the case of ipilimumab, they advocated the need for procedural fairness without political involvement. Newspapers covering specific patient stories argued for implementation on equity and severity grounds in their editorials, often quoting resource abundance in “the world’s richest country” as a rationale. Predominantly, newspaper articles portrayed disagreements by stakeholders with aspects of the decision-making process, such as price secrecy and duration, and with its outcomes.

Across the cases, we observed the common analytical themes as consistent deliberation about substantive and procedural justice as well as decision outcomes. These themes transcended A4R’s focus on fair process and political legitimacy, [3] as substantive justice and decision acceptability also appear central to stakeholders, consistent with empirical studies on priority-setting perceptions [16, 31, 32].

We found that the institutionalisation of New Methods in several media cases isolated healthcare rationing as a conversation outside the political sphere between stakeholders and decision-makers. Procedural complaints changed from healthcare rationing being portrayed as politically and technically fragmented to becoming bureaucratic and secretive. Newspaper media served as an appeals institution and communicator of allocation decisions [4], raising stakeholder action [26] and indicating transparency and broad deliberation about the rationing institution by communicating its mandate and decisions [29].

### Methodological considerations

The proposed standards for qualitative inquiry are relevance, validity, and reflexivity [57, 71]. The small sample of three cases holds relevant and valid information to elucidate the study question due to our strategic selection of cases, newspapers and newspaper articles [55].

As with all qualitative studies, the results cannot and should not be generalised as an evaluation of public participation in the Norwegian healthcare rationing debate. We acknowledge that our studied cases are among the most scrutinised and contentious allocation decisions in the recent Norwegian healthcare administration. Our results should instead be seen as a learning opportunity for stakeholders from these specific cases [71].

Another limitation is using print media as our only source of information. Public perceptions of healthcare rationing will be informed not only by print media but also by social and broadcast media as well as sources outside the media sphere [18, 21]. This implies that our findings cannot be generalised to encompass all public perceptions regarding these cases, but they are useful for a thorough examination and better understanding of the print media debate. Furthermore, we found editorial power concentration as Schibsted Publishing House owns four of the five largest newspapers in the source material. Media conglomerates may draw public attention to stories portrayed by newspapers within the same publishing house [4, 21]. We attempted to inform our study question by purposively and systematically sampling relevant cases and data from a stratum of sources covering most Norwegian newspaper readers. The application of A4R as a substantive theory to the STC method mitigated the prominence of parts of the data material not concerned with healthcare rationing, thereby enhancing the relevance of data gathering and analysis. By doing so, we judged the three cases and sampled data to hold information that is relevant and suitable for the study question [55].

A methodological concern is that STC is primarily a cross-sectional method, which makes it difficult to infer longitudinal analyses of the development of healthcare rationing. We applied STC to multiple cases and compared our findings to analyse this development. We were cautious not to assume causality between the results of our discrete cases within a continuous public debate concerning healthcare but attempted careful interpretation and discussion in development evaluation. Future research may apply longitudinal qualitative methods to assess the development of Norwegian healthcare rationing or to evaluate how the public understands the studied newspaper articles to validate our findings.

During the analysis, we remained aware of our biases and preconceptions. The author OFN led the Third Committee on priority-setting [7], whose recommendations influenced our preconceptions about the normative standards for a Norwegian system of method evaluation and priority setting, although these recommendations in turn were informed by A4R [3]. OFN’s experience provided for an insightful understanding of the media debate, and to avoid bias, OFN was excluded from analysis when his voice was found in the data material. BR holds an adjunct position at the Norwegian Institute of Public Health and is involved in some method evaluations in New Methods.

## Conclusion

To our understanding, this is the first study to use newspaper coverage to describe and assess public participation in the Norwegian healthcare rationing debate. Our findings were largely consistent with previous empirical research on priority-setting perceptions, although we noted more opposition to rationing than previously reported amongst stakeholders. We found wide media deliberation in all three cases and print media to work as an appeals institution by raising stakeholder action. Across all cases, we found strong public concern with both procedural and substantive justice as well as decision outcomes for patients.

We observed that the introduction of New Methods institutionalised the Norwegian healthcare rationing debate. The system was recognised as a legitimate distributive institution but also criticised for secrecy and patient rights violation. Public deliberation appeared isolated from politics as a conversation between decision makers and stakeholders.

## Supplementary Information

**Additional file 1.** Data search strings. Search strings for final data searches in Atekst Retriever.

## Data Availability

We purposively sampled publicly accessible newspaper articles for the analysis by using the Norwegian newspaper search engine ATEKST (www.retriever.no). All sampled articles have been saved as PDFs and are available for peer reviewers and readers per request from the first author.

## Abbreviations

RHA: Regional health authority
STA: Single technology assessment
HTA: Health technology assessment
CEO: Chief Executive Officer
A4R: Accountability for reasonableness
STC: Systematic text condensation
QALY: Quality-adjusted life-year
SMA: Spinal muscular atrophy
