# Supplementary material for: Public participation: healthcare rationing in the newspaper media
Source: BMC Health Serv Res. 2022 Mar 28;22:407. doi: 10.1186/s12913-022-07786-w (PMC8962557; doi:10.1186/s12913-022-07786-w)
Supplement: Supplementary file 1 — Additional file 1. Data search strings. Search strings for final data searches in Atekst Retriever. [file 12913_2022_7786_MOESM1_ESM.docx]

# Additional file 1: Data search strings

Search strings for final data searches in Atekst Retriever (www.retriever.no). Searches were performed on October 7^th^, 2019, and conducted for the same 9 newspapers as described in Table 1.

Search 1: “(ipilimumab OR yervoy) AND (føflekkreft OR føflekk OR melanom)”. The search produced 107 findings, of which 45 were selected for further analysis.

Search 2: “(nivolumab OR opdivo) AND (lunge or lungekreft)”. The search produced 62 findings, of which 23 were selected for further analysis.

Search 3: “(Spinraza OR nusinersen) AND (sma OR “spinal muskelatrofi”)”. The search produced 120 findings, of which 68 were selected for further analysis. Heal
